# Amm. Carb. and Kali Carb. in Morning Cough

**Published:** 1884-01

**Authors:** C. Carleton Smith

**Affiliations:** Philadelphia


### AMM. CARB. AND KALI CARB. IN MORNING COUGH.

Both Amm. carb. and Kali c. have violent cough in A. M., between three and four o'clock. The difference between the two is this—the Kali c. cough improves after breakfast, not so the Amm. c. cough; it continues with more or less severity all day. Again, Amm. c. has *choking* sensation with its chest symptoms, Kali c. has not. Also, Amm. c. has sense of suffocation; this is so bad sometimes that the patient cannot bear to have the bed-clothes brought anywhere near his mouth for fear of choking; terrible aggravation from going into warm room under Amm. c.; face gets deathly pale, and patient cannot move—must sit perfectly still. The Amm c. patient chokes in his sleep, which wakes him. Kali c. acts more on the right lung, Amm. c. on the left. Amm. c. is worse lying on left side, Kali c. o right side.

C. CARLETON SMITH.

[Kali c. has suffocating and choking cough at five A. M. as if caused by dryness of larynx. She is prevented from talking by spasm in chest, accompanied by redness in face and heat over whole body. (Jahr.)—L.]

